# Molecular diagnosis and comprehensive treatment of multiple endocrine neoplasia type 2 in Southeastern Chinese

**DOI:** 10.1186/s13053-015-0026-1

**Published:** 2015-01-20

**Authors:** Jian-Qiang Zhao, Zhen-Guang Chen, Xiao-Ping Qi

**Affiliations:** 1 Department of Head and Neck Surgery, Zhejiang Cancer Hospital, Hangzhou, 310022 Zhejiang Province China; 2 Department of Oncologic and Urologic Surgery, the 117th PLA Hospital, Wenzhou Medical University, 40 Jichang Road, Hangzhou, 310004 Zhejiang Province China

**Keywords:** Multiple endocrine neoplasia type 2, Mutation screening, RET, Prophylactic thyroidectomy, Cortical-sparing adrenalectomy

## Abstract

**Background:**

Multiple endocrine neoplasia type 2 (MEN2) is an autosomal dominant inherited endocrine malignancy syndrome. Early and normative surgery is the only curative method for MEN 2-related medullary thyroid carcinoma (MTC). In patients with adrenal pheochromocytoma, cortical-sparing adrenalectomy (CSA) can be utilized to preserve adrenocortical function.

**Methods:**

We present twenty-six of 33 MEN2 patients underwent prophylactic thyroidectomy with varying neck dissection and eight of 24 MEN2A patients with PHEO underwent adrenal-sparing surgery. Direct sequencing of entire *RET* exons was performed in all participants.

**Results:**

The *RET* mutations (p.C634Y [n = 10], p.C634R [n = 9], p.C634F [n = 2], p.C618Y [n = 8], p.C618R [n = 3], and p.M918T [n = 1]) were confirmed in 20 symptomatic patients and identified in 13 at-risk relatives (*RET* carriers). Twenty-six of 33 MEN2 patients underwent thyroidectomies with neck dissections; the mean age at the time of the first thyroid surgery and the tumor diameter of the 6 *RET* carriers was decreased compared with 20 symptomatic patients (P < 0.001 and P = 0.007, respectively), while the disease-free survival was increased (80% vs.10%, P = 0.0001). Seven *RET* carriers who were declined surgery. One of 20 symptomatic patients with MTC bone metastases after surgery received vandetanib therapy for 20 months and responded well. Additionally, 8 of 24 MEN2A patients who initially had unilateral pheochromocytomas underwent CSA, 1 developed contralateral pheochromo cytomas 10 years later, then also accepted and also agreed to a CSA. None of the patients required steroid replacement therapy.

**Conclusions:**

Based on our results, integrated *RET* screening and the pre-operative calcitonin level is an excellent strategy to ensure earlier diagnosis and standard thyroidectomy. CSA can be utilized to preserve adrenocortical function in patients with pheochromocytomas.

**Electronic supplementary material:**

The online version of this article (doi:10.1186/s13053-015-0026-1) contains supplementary material, which is available to authorized users.

## Background

Multiple endocrine neoplasia type 2 (MEN2) is an autosomal dominant inherited endocrine malignancy syndrome, with an occurrence of approximately 1 in 30 000; MEN2 is due to germline mutations in the REarranged during Transfection (*RET*) proto-oncogene (OMIM: 164761) [1],[2], which includes the following three subtypes: MEN2A (OMIM: 171400); familial medullary thyroid cancer (FMTC; OMIM: 155240); and MEN2B (OMIM: 162300) [3]. MEN2A mainly consists of MTC, adrenal pheochromocytoma (PHEO), and hyperparathyroidism (HPT). FMTC presents clinically as MTC, but four or more persons in a family or multiple generations must satisfy the diagnostic criteria for MTC without other endocrine gland neoplasm features [3],[4]. MEN2B-related MTC has a higher invasiveness than MEN2A or FMTC, and often accompanies PHEO, mucosal and gastrointestinal ganglioneuromas, corneal nerve thickening (CNT), and Marfan’s body habitus [5]. Of patients with MEN2, 95%-100% will develop MTC, which is usually asymptomatic at an early stage; clinical symptoms usually present with lymph node metastasis (LNM) in the third or fourth decade of life. Distant metastases of MTC are the major cause of death [5]-[7].

MEN2 patients can be diagnosed early through systematic pedigree investigation and *RET* proto-oncogene screening [3]-[5]. In the vast majority of cases, > 70 *RET* mutations accumulate that are related to MEN2, and > 98% of *RET* mutations are located in exons 5, 8, 10, 11, and 13–16 [4]. In 2009, the American Thyroid Association (ATA) performed a timing prophylactic total thyroidectomy (TT) procedure based on a model that utilized the following genotype-phenotype correlations to stratify mutations into four risk levels: ATA-A → D, A < B < C < D. It was recommended that ATA-B and -C level mutation carriers should be considered for prophylactic TT, which should be performed before 5 years of age. However, it has been shown that family members with the same *RET* mutation may have different clinical phenotypes or offspring might exhibit earlier MTC phenotypes [4],[5].

In recent years, combined detection of *RET* mutations and pre-operative calcitonin (pre-Ct) levels have been considered for comprehensive decision-making regarding the timing or surgical extent of prophylactic TT [8]-[11]. Moreover, phase III clinical trials have demonstrated that one oral drug (such as vandetanib), which is a small molecular multi-targeted tyrosine kinase inhibitor, provides a new therapeutic choice for advanced hereditary MTC patients whose disease cannot be resected or who present with metastatic lesions [12]. Because most of the MEN2A-related PHEO presentations are benign with bilateral multiple onsets, it is currently preferred to perform laparoscopic cortical-sparing adrenalectomy (CSA) [5],[13],[14].

In the present study, we report 33 MEN2 cases derived from 9 unrelated MEN2 families residing in southeastern China. Our aim was to analyze the feasibility of prophylactic TT based on predictive integrated screening of *RET* and pre-Ct levels. We also observed and evaluated the efficacy and safety of vandetanib in the treatment of one advanced case of MEN2A-MTC, and determined the clinical significance of managing unilateral CSA for MEN2A-related PHEO.

## Methods

### Patients

Nine MEN2 families from Zhejiang Province of southeastern China were recruited. All patients were diagnosed and treated at the Zhejiang Cancer Hospital or the 117th PLA Hospital between August 1989 and January 2013. Systematic family screening was performed between 2005 and 2013, and comprehensive medical data, including clinical profiles, surgical procedures, pre- and post-operative biochemical data, and follow-up records were studied. The patients diagnosed before 1993 were submitted to *RET* genetic analysis at the time of family screening. All participants and/or their legal guardians provided written informed consent, as required by the institution’s Ethical Committee.

### Clinical approach

All *RET* gene mutation carriers underwent biochemical and imaging examinations to ascertain thyroid, parathyroid, and adrenal gland involvement. The biochemical examination included Ct by chemiluminescence assay (normal male, <8.4 ng/L; and female, <5.0 ng/L), parathyroid hormone (PTH) by electrochemical immunoassay, carcinoembryonic antigen by chemiluminescence assay, catecholamines, 3-methyl-4-hydroxy mandelic acid by radioimmunoassay, serum calcium by the arsenazo III method, and serum phosphate by UV molybdate. The imaging examinations included thyroid ultrasound/CT, adrenal gland and parathyroid gland nuclear magnetic resonance (MRI), emission computed tomography (ECT), and/or PET-CT scan assessments, if indicated.

### Genetic testing

Genomic DNA was isolated from the peripheral blood of 86 family members. The coding region of *RET* exons 5, 8, 10, 11, and 13–16 was amplified and sequenced in sense and antisense directions with an ABI Prism 3730 automatic sequencer (Perkin-Elmer, USA).

### Follow-up management

All patients were followed for evaluation of tumor recurrence and metastasis after surgery. Since 2005, the standard follow-up for MTC has consisted of determining the Ct level, plasma calcium level (every 6 months), and during the post-operative period under conditions in which the Ct level is abnormal, a detailed imaging examination may be considered (yearly). For MEN2A-related PHEO patients, biochemical and imaging examinations were repeated every 6–12 months, and life-time follow-up was then required. For *RET* mutation carriers without PHEOs, after initial screening at 20 years of age, repeat biochemical and imaging examinations were performed every year to screen for PHEOs and HPT [5].

### Statistical analysis

All data were analyzed with SPSS (version 12.0) software. We carried out univariate analysis using the Fisher’s exact test. Student’s t-test was used to analyze differences between mean values.

## Results

### Clinical and diagnostic data

**Figure 1:**
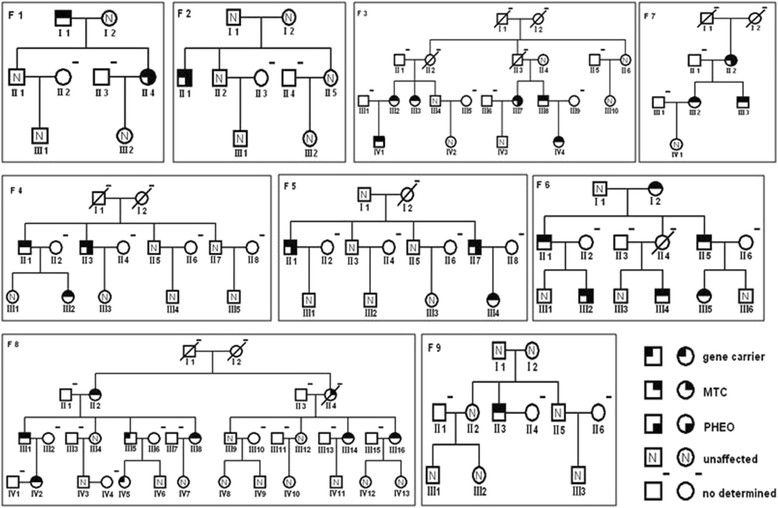
Genealogy of seven MEN2A families (F1-F7), one FMTC family (F8) and one MEN2B family (F9).

The 33 MEN2 patients consisted of 9 index-cases (7 males and 2 females), 11 clinical patients (5 males and 6 females) and 13 asymptomatic carriers (4 males and 9 females). The mean age at the time of diagnosis was 38.6 ± 10.7 years (range, 23–58 years), 38.8 ± 16.6 years (range, 20–65 years), and 30.0 ± 18.5 years (range, 9–78 years), respectively. All patients derived from 9 unrelated MEN 2 families and harbored 1 of 6 heterozygous missense mutations; 7 families had MEN2A (p.C634Y [n = 3], p.C634R [n = 2], p.C634F [n = 1], p.C618R [n = 1]), 1 family had FMTC (p.C618Y [n = 1]), and 1 family had MEN2B (p.M918T [n = 1]). Of the 33 MEN2 patients, 32 (97%) were diagnosed with MTC (31) or C-cell hyperplasia (1) and 8 (33.3%) were diagnosed with PHEO (Table 1 and Figure 1). None of the 33 MEN2 patients had consistently evidence of any co-occurring related MEN2 clinical symptoms (pruritic cutaneous lichen amyloidosis or Hirschsprung’s disease), or HPT (serum PTH, Calcium or PTH/calcium rates, and serum phosphate levels ranged in all normal), except one MEN2B patient (F9-II3) had a broad spectrum of pathognomonic nonendocrine abnormalities: a Marfanoid body habitus, tongue mucosal neuromas, lips hypertrophy and CNT.

**Table 1:** Clinical and molecular features of nine MEN2 families

Family	Numbers of patients	Sex ratio (M/F)	Age range (years)	*RET*mutation	MTC age at onset (mean years)	PHEO numbers/Age at diagnosis (mean years)
MEN2A						
F1	2	1/1	28-58	p.C634F	43.0	1/26
F2	1	1/0	35	p.C634Y	35.0	1/35
F3	6	2/4	9-44	p.C634Y	30.2	1/36
F4	3	2/1	18-44	p.C634Y	27.3	1/47
F5	3	2/1	19-32	p.C634R	24.7	2/40.5
F6	6	4/2	19-65	p.C634R	33.5	1/21
F7	3	1/2	21-36	p.C618R	27.3	1/40
FMTC						
F8	8	2/6	24-78	p.C618Y	45.3	-
MEN2B						
F9	1	1/0	33	p.M918T	33	-

### Genogram MTC

Of the 33 MEN2 patients, 32 (97% [20 symptomatic patients including 9 index-cases and 11 clinical patients, and 12 asymptomatic carriers]) who were diagnosed with MTC or C-cell hyperplasia (CCH) clinically or based on pathologic evaluation, the mean diagnostic age was 34.6 ± 16.1 years; 1 *RET*-carrier (F8-III5) had no abnormalities.

Twenty symptomatic patients were admitted to the hospital for neck masses or neck pain and diagnosed with MTC before clinical screening. Thirteen of 20 (65%) patients were subjected to sub-standard thyroid surgery: 8 patients underwent interval (at least 2 times) TT and bilateral VI region lymph node dissection [B-LND (VI)] or modified bilateral cervical lymph node dissection (MBND); 3 patients underwent bilateral sub-TT and/or MBND; and 2 patients underwent unilateral TT and B-LND (VI) with MBND. The other 7 patients were accepted for standard thyroid surgery, as follows: 5 patients underwent TT plus B-LND (VI) plus MBND; and 2 patients underwent TT plus B-LND (VI). The histopathologic results showed that 20 patients had bilateral MTC (1 patient with T2N0M0 and 19 patients with T1N1bM0 ~ T3N1bM0; Table 2). The median time of follow-up thus far is 115.6 ± 90.4 months (range, 21–384 months). The results of post-Ct levels were normal in 2 patients (10% [T2N0M0 and T1N1bM0]) and elevated in 18 patients (90%). Moreover, 2 of 20 patients (F6-II5 and F4-II1) had elevated Ct levels > 2000 ng/L. One patient (F6-II5), a 39-year-male-old with T3N1bM0, underwent surgery successively 4 times and eventually exhibited multiple bone metastases based on ECT and MRI examinations 11 years after the initial surgery. Vandetanib (ZD6474, Zactima™) was administered orally at an initial dose of 300 mg/d; bone pain resolved and the Ct levels decreased from 8755.3 ng/L to 873.6 ng/L after 2 months. Eight months later, the patient appeared uncomfortable and distressed. An electrocardiogram showed Q-T interval prolongation and incomplete right bundle branch block. Subsequently, the dose of vandetanib was decreased from 300 mg to 200 mg once a day for 20 months (until the present). The Ct level increased to 5869.2 ng/L during therapy, while repeated PET-CT examinations failed to show any obvious advance bone metastatic lesions. The patient developed erythema after vandetanib therapy, but had no pruritus, headaches, nausea, or reduction in the number of white blood cells. The other patients (F4-II1), a 50-year-male-old with T2N1bM0, declined further therapy and has survived with the tumor *in situ* for 19 years (until the present; Table 2).

**Table 2:** Characteristics of symptomatic patients and*RET*-mutation carriers

	Total (n = 33) N (%)	Symptomatic patients (n = 20) N (%)	Mutation carriers (n = 13) N (%)	*P*value
**Female patients**	16 (48.5%)	7(35%)	9 (69.2%)	
Genotype				
p.C634F/Y/R	21	12	9	
p.C 618Y/R	11	7	4	
p.M 918 T	1	1	0	
**Surgery**				
Numbers of patients operated	26	20	6	
Age at first surgery, mean (years)	34.6 ± 16.1	38.7 ± 13.9	20.7 ± 2.9	<0.001
TT with central and lateral LND	14	13	1	
TT with central LND	5	2	3	
TT without LND	2	0	2	
Inappropriate	5	5	0	
Histology				
Tumor diameter (cm, mean)	2.2 ± 1.2	2.6 ± 1.0	0.7 ± 0.4	0.007
CCH	1	0	1	
MTC	25	20	5	
Lymph node metastasis				
N0	6	1	5	
N1	20	19	1	0.0001
Distant metastasis				
M0	23	18	5	
M1	2	2	0	
Disease-free survival	6	2	4	0.0001

Thirteen asymptomatic *RET* carriers were confirmed by clinical family screening, 12 of whom had elevated pre-Ct levels; 6 patients underwent prophylactic TT (n = 2), prophylactic TT plus B-LND (VI; n = 3), or prophylactic TT plus MBND (n = 1). The histopathologic examination revealed MTC in 5 patients (4 patients with T1N0M0 and 1 patient with T1N1bM0) and 1 26-year-old patient with CCH (F8-IV5; Table 3). The median time of follow-up thus far has been 19.6 ± 3.2 months (range, 17–26 months) and the results of post-Ct levels were normal in 5 patients. Unfortunately, a 21-year-old male (F6-III4) with T1N1bM0 still has an elevated Ct level. In addition, the remaining 7 patients declined/awaited surgery, 6 of whom had progressively increased Ct levels, and only a 53-year-old male (F8-III5) has a normal Ct level and no thyroid nodules based on imaging (Table 3).

**Table 3:** Characteristics of 13*RET*-mutation carriers

Patient/Gender	*RET*mutation	Age	Pre-Ct (ng/L)	CEA (ng/ml)	Tumor size* (cm): L/R	Operative procedure	Histology	pTNM	LNM	Post-Ct (ng/L)	Interval time (years)
F3-III8/F	C634Y	41	1744.0	57.9	1.4/3.2	Reject operation	-	-	-	-	18
F3-III9/F	C634Y	37	206.0	12.2	1.0/0.7	Reject operation	-	-	-	-	18
F3-IV1/M	C634Y	22	11.8	1.8	-/0.3	Reject operation	-	-	-	-	18
F3-IV4/F	C634Y	9	9.8	0.7	0.3/0.2	Reject operation	-	-	-	-	18
F4-III2/F	C634Y	18	118.0	6.8	1.0/1.0	TT + B-VI	MTC	T1N0M0	0/5	<2.0	17
F5-III4/F	C634R	19	24.3	2.9	0.3/0.2	TT	MTC	T1N0M0	NO	<2.0	17
F6-III2/M	C634R	21	20.9	2.7	0.5/0.7	TT + B-VI	MTC	T1N0M0	0/3	<2.0	26
F6-III4/M	C634R	21	1769.0	119.6	1.1/1.0	TT + B-VI + R-LND	MTC	T1N1bM0	20/59	975.0	23
F6-III5/F	C634R	19	71.4	3.3	0.5/1.0	TT + B-VI	MTC	T1N0M0	0/1	<2.0	26
F7-III2/F	C618R	26	487.6	25.9	2.0/2.5	Reject operation	-	-		-	17
F8-II2/F	C618Y	78	1758.0	106.0	3.0/3.5	Reject operation	-	-		-	19
F8-III5/M	C618Y	53	<2.0	2.2	−/−	Awaiting surgery	-	-		-	19
F8-IV5/F	C618Y	26	5.8	2.0	−/−	TT	CCH	-	NO	<2.0	19

**Note:** Ct (basal serum Ct): normal <8.4 ng/L for male and <5.0 for female by FACLIA ; CEA (carcinoembryonic antigen): normal ≤ 5.0 ng/ml; Pre-Ct, pre-surgical Ct; Post-Ct, post-surgical Ct; TT, total thyroidectomy; B-VI, bilateral level VI lymph node dissection; R/ L -LND, right/ left lymph neck dissection.

*Tumor size was measured by ultrasonography.

The asymptomatic *RET* carriers who underwent surgery benefited greatly from genetic screening and were a significantly younger age at the time of surgery and had a smaller tumor diameter compared with symptomatic patients (*P* < 0.001 and *P* = 0.007, respectively). One *RET* carrier had lymph node metastasis compared with 19 of 20 symptomatic patients (*P* = 0.0001). The disease-free survival (DFS) increased in the asymptomatic *RET* carriers who underwent surgery compared to the symptomatic patients (80% vs. 10%, *P* = 0.0001; Table 2).

### PHEO

Of 24 MEN2A patients, 8 presented with PHEOs, including 5 males and 3 females (7 p.C634Y/R/F and 1 p.C618R *RET*-mutations) (Table 4). The mean age at the time of diagnosis was 35.8 ± 9.2 years (range, 21–47 years). Among 8 PHEO patients, 5 were symptomatic and 3 were asymptomatic. The asymptomatic patients were diagnosed during systematic pedigree analysis. In addition, all 8 PHEO patients initially presented with unilateral disease. Unilateral CSA was initially performed, and 4 of 8 patients underwent a laparoscopic technique. The average maximum diameter of the PHEOs was 3.9 ± 2.5 cm (range, 2.3-10.0 cm), and all of the PHEOs were located within the adrenal glands. One patient (F5-II1) relapsed after initially developing a contralateral tumor after 10 years, and underwent CSA on the opposite side (bilateral CSA). After surgery, none of the 8 patients required steroid replacement therapy. The median time of follow-up thus far has been 88.3 ± 84.1 months (range, 8–252 months). There were no surgical complications, such as hypoadrenalism, Addison’s crisis, local recurrence, or distant metastasis. Interestingly, the 58-year-old father (F1-I1) of a *RET* carrier (F1-II4) with unilateral PHEO and MTC in the F1 family had presented with MTC as the only clinical symptom, but no evidence of a PHEO. Furthermore, in the F6 family, the first (I) and second (II) generation also had the only clinical manifestations of MTC, while only one patient in their offspring (III-2) had evidence of a PHEO.

**Table 4:** Clinical and pathological features of 8 MEN2A patients with pheochromocytoma

Patient	Gender	*RET*mutation	Age at diagnosis PHEO	Temporal relation of PHEO to MTC	NE/E/DA (ng/L)	Tumor (size, cm)	Operative procedure	Follow-up
F1-II4	F	C634F	26	Preceding	246.0/36.3/61.5	L, 3.3	Left OPE	163
F2-II1	M	C634Y	35	Concomitant	399.0/48.2/43.0	R, 2.5	Right OPE	74
F3-III7	F	C634Y	36	Preceding	827.5/106.0/88.5	R, 4.0	Right OPE	71
F4-II3	M	C634Y	47	Succeeding	657.0/144.2/40.0	L, 2.3	Left LPE	8
F5-II1	M	C634R	34	Succeeding	1305.0/1270.3/243.6	L, 10 ; R, 3.5	heterochronous bilateral OPE	252
F5-II7	M	C634R	47	Succeeding	667.7/92.5/78.3	R, 2.9	Right LPE	12
F6-III2	M	C634R	21	Concomitant	69.9/12.6/10.2	L, 3.5	Left LPE	24
F7-II2	F	C618R	40	Succeeding	681.2/89.4/67.0	L, 2.9	Left LPE	102

**Note:** OPE, open PHEO excision; LPE, laparoscopic PHEO excision; NE (norepinephrine): normal, 0 ~ 600 ng/L; E (epinephrine): normal, 0 ~ 100 ng/L; DA(dopamine): normal, 0 ~ 100 ng/L.

### Follow-up management

Seven asymptomatic *RET* carriers were strictly monitored and presented with gradually increasing Ct levels, but did not undergo surgery. The other 26 patients received thyroid hormone replacement therapy lifetime, and were given calcium and vitamin D3 as preventive supplements at least 1 year after surgery. Moreover, thyroid, adrenal, and parathyroid function was normal, with no evidence of complications, such as tetany.

### RET germline variants

All 33 *RET* mutation-positive patients (consisting of 20 symptomatic patients and 13 asymptomatic *RET* carriers) harbored 1 of 6 heterozygous missense mutations within exons 10 (p.C618Y [n = 8] and p.C618R [n = 3]), 11 (p.C634Y [n = 10], p.C634R [n = 9], and p.C634F [n = 2]), 16 (p.M918T [n = 1]). It was surprising to find that in the F2 and F9 families, only proband (F2-II1 and F9-II3) had the p.C634Y or p.M918T mutation, while his consanguineous parents and brothers were shown not to harbor the mutations or MEN2A/MEN2B-related manifestations, respectively (Table 1).

## Discussion

In this study, the rate of occurrence of MEN2-related MTC/CCH, PHEO and the mean diagnostic age was similar to previously reported data [15],[16]; however, MEN2-related HPT was not identified in the current study, which was different from Zhou *et al*. [17] reported that the prevalence of Chinese MEN2-related HPT was 10.8%, which is also lower compared with Western countries (20%-30%) [4]. The difference may be related to regional disparities, limited sample size and racial distribution.

Post-operatively, 17 of 20 (85%) symptomatic patients presented with residual MTC, metastases, and/or a recurrence. Five of 6 (83.3%) asymptomatic *RET* carriers underwent prophylactic TT had no abnormalities (*P* < 0.05). MEN2 had an age-related penetrance and metastasis of MTC with a variable intensity of expression. For the 10% of MEN2-related MTC patients with cervical LNM who were cured by TT and extensive lymph node excision, early standard surgery based on diagnostic *RET* testing might distinctly improve the cure rate of MEN2-related MTC [3]-[5],[18],[19]. In addition, even the same family has significant variability in the clinical expression of disease and development of MTC [for example, F8-III5, F8-IV5 and F8 other 6 with p.C618Y]. This may occur by the accumulation of genetic changes that have a dominant effect on the mutant allele or other affected factors [20],[21].

In 2009, at the 9th meeting of the European Thyroid Association and Cancer Research Network (ETA-CRN), there were comments reported to the 2009 ATA guidelines that pointed out that timing of prophylactic TT should be at best synthetically decided by combining the results of both *RET* gene testing and the ongoing pre-Ct level [9]. The timing of TT in *RET* carriers with a negative Ct level can be personalized and safely planned when the stimulated Ct becomes positive. Intrathyroid MTCs were found when the pre-Ct level was <60 ng/L, and individualization of surgery in *RET* carriers with normal pre-Ct levels may be avoided B-LND (VI) [8],[10]. QI *et al*. [11] also reported that MTC remained located within the thyroid at low concentrations of Ct (<71.4 ng/L) in 17 asymptomatic *RET* carriers from the Chinese Han nationality. Here, the 3 patients (F4-III2, F6-III2, and F6-III5) might have avoided prophylactic B-LND (VI). More recently, Pelizzo *et al*. [22] demonstrated that *RET* carriers with normal basal Ct levels undergoing prophylactic TT after testing positive for stimulated Ct could not always be cured, particularly in 12% of all the level ATA-B/C *RET* mutations cases. The threshold for a definitive cure of MEN2-related MTC still needs to be empirically determined. The 7 asymptomatic *RET* carriers declined surgery or chose a watchful waiting approach, which offered us the unique opportunity to observe the natural history of MEN2-related MTC. Thus, it is important to increase patient awareness to avoid treatment delay. Furthermore, our observation was also similar to reports detailing the use of vandetanib for advanced genetic MTC, and the Q-T interval prolongation of drug toxicity was remarkable [12],[23],[24].

Moreover, in the early stages of research, the MEN2A family (F6, p.C634R) was mistaken for the FMTC family; however, it was soon shown that the F6-III2 family accompanied PHEOs. FMTC is associated with mutations in codon 634 and is most commonly identified as p.C634Y, and almost never p.C634R [25]. It has been previously suggested that it is important to use clinical screening and deeper genotype-phenotype correlations in intact pedigree analysis including long-term follow-up, which may avoid missed diagnoses of PHEOs and/or HPT and other endocrine adenomas besides the diagnosis of MTC (FMTC). It has also been suggested that family members with the same *RET* mutation may display different clinical phenotypes, and the offspring may display earlier PHEO phenotypes [4]. Our findings also implied that MEN2-related PHEO occurrence may be affected by modifying factors from parents or the environment, and even by other mechanisms, such as errors in chromosomal replication during cell division or a second somatic mutation [4],[26]. Additionally, sufficient pre-operative preparation before PHEO is necessary and preferential PHEO excision favors the avoidance of burst hypertensive crisis caused by PHEO during MTC or other non-PHEO surgical procedures or even death [4],[5],[26]. Unilateral MEN2A-related PHEO patients usually show contralateral PHEO over a period of 10 years and malignant MEN2A-related PHEO is rare (0.4%-5.0%) [4],[27],[28]. Based on a multi-institutional study, Castinetti *et al*. [28] recently reported 563 MEN2-related PHEO patients; recurrence of PHEOs in the same adrenal gland after CSA was only reported in 4 (3%) of 153 operated glands or in 4 (4%) of 114 patients after 6–13 years. Moreover, 47 of 82 (57%) patients with bilateral PHEO who underwent CSA did not become steroid-dependent. The CSA is a feasible surgical strategy for MEN2-related PHEOs, which may avoid or postpone the need for steroid replacement therapy and the associated risk of an Addisonian crisis [14],[28]. The post-operative MEN2-related PHEOs should undergo follow-up, and monitored long-term for timely diagnosis and treatment options to prevent or reduce clinical risks [4],[5]. In this study, our results also showed that initially unilateral CSA was technically safe and feasible, and may reduce steroid replacement therapy after a second/contralateral CSA.

The *de novo* occurrence rate of MEN2A/FMTC was 5.6%-9.0% [29], while in MEN2B > 90% of cases presented with *de novo* germline *RET* mutations [30]. In the current study, in 2 (F2 [p.C634Y] and F9 [p.M918T]) of 9 families (Figure 1), only 2 probands harbored mutations; all the other consanguineous family members had no p.C634Y or p.M918T mutations, which suggested that both patients had *de novo* mutations or that the mutations existed in a parental germ cell mosaicism [31]. The age at which the p.M918T mutation was diagnosed in our patient was advanced compared with the mean age (13 ± 9.3 years) reported by Brauckhoff *et al*. [30]. Thus, awareness of the clinical relevance of nonendocrine manifestations, timely detection of *RET* gene and pre-Ct facilitate an early diagnosis and normalize surgery to improve the long-term outcome of MEN 2B.

## Conclusions

In summary, systematic evaluation of MEN2 patients by means of integrated screening of *RET* and pre-Ct levels and implementation of individualized prophylactic TT favors a reduction in surgical complications and improves DFS. For advanced MTC, vandetanib provides novel treatment options. In patients with PHEOs, CSA can be utilized to preserve adrenocortical function.

## Authors’ original submitted files for images

Below are the links to the authors’ original submitted files for images. Authors’ original file for figure 1
