# Supplementary figures and images for: Molecular diagnosis and comprehensive treatment of multiple endocrine neoplasia type 2 in Southeastern Chinese
Source: Hered Cancer Clin Pract. 2015 Jan 20;13:5. doi: 10.1186/s13053-015-0026-1 (PMC4307225; doi:10.1186/s13053-015-0026-1)

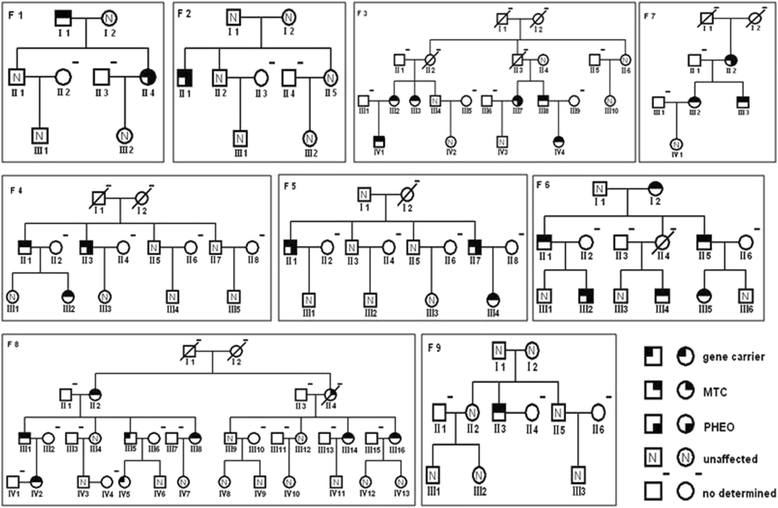

Supplement: Supplementary file 1 — Authors’ original file for figure 1 [file 13053_2015_26_MOESM1_ESM.gif]
